# Interim Guidance for Urodynamic Practice during COVID-19 Pandemic

**DOI:** 10.1590/S1677-5538.IBJU.2020.0671

**Published:** 2020-11-18

**Authors:** André Avarese Figueiredo, Ailton Fernandes, Alexandre Fornari, Aleia Faustina Campos, Mario Henrique Tavares Martins, Carolina Mayumi Haruta, Silvio Henrique Maia Almeida, Luís Gustavo Morato de Toledo, Daniel Carlos Moser, André Luiz Farinhas Tomé, Márcio Augusto Averbeck, Cristiano Mendes Gomes

**Affiliations:** 1 Universidade Federal Departamento de Cirurgia Juiz de ForaMG Brasil Departamento de Cirurgia, Universidade Federal de Juiz de Fora, MG, Brasil; 2 Universidade do Estado do Rio de Janeiro Departamento de Urologia Rio de JaneiroRJ Brasil Departamento de Urologia, Universidade do Estado do Rio de Janeiro, Rio de Janeiro, RJ, Brasil; 3 Santa Casa de Porto Alegre Porto AlegreRS Brasil Serviço de Urologia, Santa Casa de Porto Alegre, Porto Alegre, RS, Brasil; 4 Universidade de São Paulo Faculdade de Medicina Hospital das Clínicas São PauloSP Brasil Divisão de Doenças Infecciosas do Hospital das Clínicas da Faculdade de Medicina da Universidade de São Paulo, São Paulo, SP, Brasil; 5 Hospital Universitário de Sergipe Divisão de Urologia SergipeSE Brasil Divisão de Urologia, Hospital Universitário de Sergipe - HU-UFS - EBSERH, Sergipe, SE, Brasil; 6 Instituto Materno Infantil de Pernambuco Unidade de Disfunção do Assoalho Pélvico Feminino RecifePE Brasil Unidade de Disfunção do Assoalho Pélvico Feminino, IMIP, Recife. PE, Brasil; 7 Universidade Estadual de Londrina Disciplina de Urologia LondrinaPR Brasil Disciplina de Urologia, Universidade Estadual de Londrina, Londrina, PR, Brasil; 8 Faculdade de Ciências Médicas da Santa Casa de São Paulo São PauloSP Brasil Disciplina de Urologia, Faculdade de Ciências Médicas da Santa Casa de São Paulo, São Paulo, SP, Brasil; 9 Instituto D'Or de Pesquisa e Educação São PauloSP Brasil Instituto D'Or de Pesquisa e Educação, São Paulo, SP, Brasil; 10 Hospital Ana Costa SantosSP Brasil Serviço de Urologia, Hospital Ana Costa, Santos, SP, Brasil; 11 Hospital Moinhos de Vento Porto AlegreRS Brasil Serviço de Urologia, Hospital Moinhos de Vento, Porto Alegre, RS, Brasil; 12 Universidade de São Paulo Faculdade de Medicina Divisão de Urologia São PauloSP Brasil Divisão de Urologia, Faculdade de Medicina da Universidade de São Paulo, São Paulo, SP Brasil

**Keywords:** COVID-19 diagnostic testing [Supplementary Concept], Lower Urinary Tract Symptoms, Coronavirus

## INTRODUCTION

COVID-19 pandemic has caused profound changes in medical practice globally. Regular patient care has been dramatically affected since the focus of both the public and private health systems being shifted to the management of patients with COVID-19 (1, 2). Avoidance of non-emergency treatments and medical procedures have been recommended worldwide and are in effect in Brazil since the beginning of March/2020 (3).

Social distancing has been introduced more than four months ago and is plainly effective throughout the country, with no signs that it will be relaxed in the next weeks. A recent study showed a dramatic reduction in elective patient visits and surgical procedures in the whole country (4). Given the continental dimensions and significant geographical and socioeconomic heterogeneity of the country, the pandemic impact has also been heterogeneous across different regions in Brazil (5). In addition, governmental statemnts supporting economy reopening and easing social distancing are also different based on parameters like the infection rate, hospital bed occupancy and testing and isolation capacity. Based on a system with progressive phases, different cities and states in Brazil are in distinct stages of restrictions for non-essential activities.

Another important element that may influence patients’ drive to search for medical evaluation and treatment of non-emergency conditions is the psychological impact of quarantine and its fatigue (6). People are becoming more lenient toward unnecessary trips and outside activities after being quarantined for almost four months since the pandemic's outset and less adherent to authorities’ recommendations.

More importantly, specialists and patients are becoming increasingly worried about the potential harm and impact in quality of life caused by deferring medical evaluation and treatment for a long time (6). In functional urology, it includes lower urinary tract dysfunction associated with benign prostatic hyperplasia, neurogenic lower urinary tract dysfunction, overactive bladder, male and female stress urinary incontinence and other conditions.

In this scenario, we observe a gradual reactivation of urodynamic practices moved by the growing demand for evaluation. Guidance must be provided about priority changes regarding urodynamic indications and on recommendations for protective measures to minimize the risk of infection with the SARS-COV-2 virus for patients and health care professionals.

This article reviews the most recent guidelines focused on urology and urodynamic practice during the COVID-19 pandemic from the International Continence Society (7), European Association of Urology (8, 9) and American Urological Association (10, 11). Our present document is based on these guidelines and represents the recommendations of the Brazilian Society of Urology (SBU - Sociedade Brasileira de Urologia) regarding the practice of urodynamics during the COVID-19 pandemic in Brazil. We provide guidance based on clinical priority and the state of restrictions for medical practice and social/economic activities. We also provide recommendations for reducing the risk of COVID-19 infection for patients and health care professionals when urodynamics is performed.

A. Urodynamic indications during COVID-19 Pandemic

Practitioners must balance the need to provide necessary services while minimizing risk to patients and health care professionals. The potential for patient harm if care is deferred must be considered when making decisions about ordering urodynamic tests in a similar extent than when making decisions about providing elective procedures, surgeries, and non-urgent outpatient visits.

DO NOT perform urodynamic evaluation in suspected or confirmed SARS-COV-2 active infections. In patients who have already had the infection, urodynamics MAY BE PERFORMED after 14 days of hospital discharge (moderate or severe illness) or after 14 days of the onset of symptoms (mild illness), if the patient is totally asymptomatic (8). If in doubt, consider performing targeted SARS-CoV-2 testing of asymptomatic patients. Depending on guidance from local and state health authorities, testing availability, and how rapidly results are available, facilities can consider implementing pre-admission or pre-procedure diagnostic testing with authorized nucleic acid or antigen detection assays for SARS-CoV-2 (9).

According to the local government flexibility of medical practice during the COVID-19 pandemic and the priority of urodynamic indications (7), three situations were analyzed (Table-1):

**Table 1:** Recommendations for urodynamic practice according to local government restrictions and risk classification during COVID-19 pandemic.

LOCKDOWN	
Do not perform urodynamics	
**HIGH RESTRICTIONS FOR MEDICAL PRACTICE AND SOCIAL/ECONOMIC ACTIVITIES**	
Urodynamics may be performed considering adaptations to the COVID-19 pandemic.	
**Prioritize high risk patients:**	
Risk for upper urinary tract deterioration In evaluation for bladder reconstructive surgery or kidney transplantation. Urinary retention or other complications	
**Defer Urodynamics in low risk patients:** Stress urinary incontinence, overactive bladder, non-neurogenic male and female LUTS with low risk for upper urinary tract deterioration
**LOW RESTRICTIONS FOR MEDICAL PRACTICE AND SOCIAL/ECONOMIC ACTIVITIES**	
Urodynamics may be performed for all guideline-recommended conditions

**LUTS** = Lower Urinary Tract Symptoms

- Lockdown.
  If your city is under lockdown and only emergencies and urgent medical procedures are allowed, DO NOT PERFORM urodynamic evaluation.
- High restrictions for medical practice and social/economic activities
  This is the present situation of most cities in Brazil. Urodynamics MAY BE PERFORMED considering adaptations to the COVID-19 pandemic. High risk patients should be prioritized and urodynamics should be deferred in low risk patients. The following risk stratification is based on international recommendations (7, 11) and our expert opinion:
  High risk patients:
  – Patients under risk for upper urinary tract deterioration: those with spinal cord injury, spinal dysraphism, multiple sclerosis and other neurological conditions known to generate increased bladder pressure.
  – Patients being considered for a bladder reconstructive surgery such as bladder augmentation, urinary diversion, or kidney transplantation.
  – Patients with urinary retention or other complications (i.e., hydronephrosis, bladder stones, diverticulum) when the confirmation of bladder outlet obstruction is considered critical for patient management.
    Low risk patients:
  – Patients with stress urinary incontinence, overactive bladder and others non-neurogenic conditions associated with LUTS in men and women that carry a low risk for upper urinary tract deterioration.
- Low restrictions of medical practice and social/economic activities

Urodynamics MAY BE PERFORMED for all guideline-recommended conditions, always balancing the need to provide necessary evaluation while minimizing risk to patients and health care professionals.

Recommendations for COVID-19 prevention and control when performing urodynamics: minimizing risks for patients and health care professionals.

**Figure 1:**
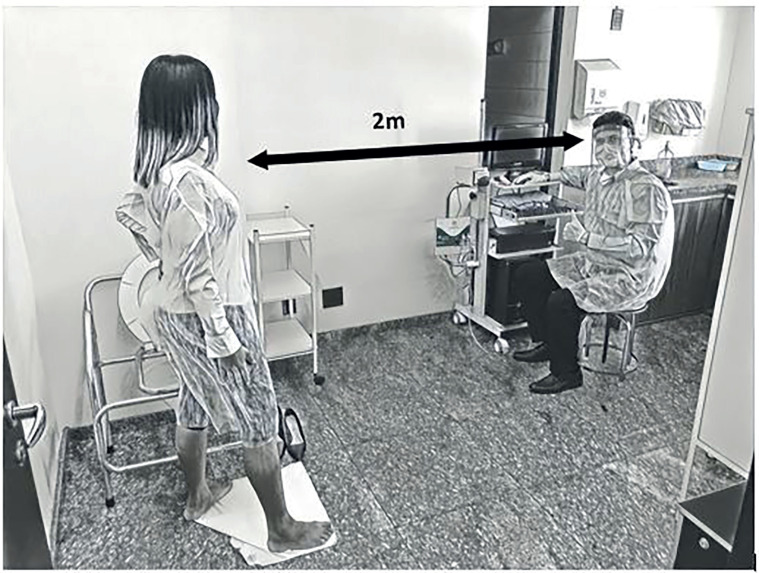
Adaptations for urodynamic performance include: single-use gloves, apron, mask; and eye protection for staff; mask for the patient and maintenance of 2 meters distance between examiner and patient; the patient is standing for stress incontinence maneuvers (straining) with a pad placed on the floor to facilitate observation of urine leakage from distance.

Our present recommendations are based on statements issued by international societies (7–11) in order to reduce the risk of COVID-19 infection for patients and health care professionals. Additional prevention and control practices are to be used along with the standard proceedings of the urodynamic tests. They are summarized in Table-2 and include measures to be implemented before and during the urodynamic test (Figure-1).

**Table 2:** Adaptations for Urodynamic performance during COVI-10 pandemic.

**Before Urodynamics**	**Phone Contact to:** Evaluate COVID-19 symptoms or contact Collect patient history Inform about mask use and companions’ restriction. Schedule patients with longer interval Standard Urodynamic rooms can be used Standard ventilation and air conditioning
**Urodynamics Day**	Establish a route to the Urodynamic room to minimize contact Provide conditions for frequent hand hygiene Screen for COVID-19 symptoms Temperature measurement Assure mask use all times Keep personal distance of 2 meters
**PPE**	**Patients and companions** Always wear a mask **Examiner and staff** Single-use gloves, aprons, and mask Face visor or protection glass disinfected with 70% liquid alcohol
**Stress Incontinence Test**	Favor standing or squatting position Favor Valsalva maneuver instead of cough If needed, cough with protection

**PPE** = Personal Protective Equipment

- Prior to the exam
  Make a detailed phone contact to:
  – Investigate if the patient has symptoms of COVID-19 (i.e. fever, cough, myalgia or fatigue (12)) close to the day he/she is scheduled to the urodynamic test or had contact with someone who had the disease in the last two weeks. Urodynamics must be postponed in any of these circumstances and the patient instructed to seek proper COVID-19 evaluation.
  – Ensure a comprehensive patient history, avoiding history taking during personal contact to minimize exposure time for patients and staff.
  – Reinforce the need to wear a face mask at all times.
  – Restrict the presence of companions and the need for them to also comply with prevention measures.
  Schedule patients with longer intervals to avoid contact between patients and their companions and ensure physical distancing. Standard urodynamic rooms can be used, since a negative pressure environment is not required, and positive pressure rooms are not recommended (7, 13). Urodynamic tests are not considered to be aerosol-generating procedures and there is no need for full air change in the room between patients and no need to change ventilation or air conditioning in the room (7, 9, 14).
  Ensure that environmental cleaning and disinfection procedures are properly performed by applying disinfectants for use against SARS-CoV-2 that are recommended by health authorities. This should be done at frequent intervals in the waiting area and all rooms of the urodynamic unit and specially in the urodynamic room after each test (7).
  On the day of the exam, establish a route for patients and companions once they arrive at the hospital or clinic to minimize contact with other visitors. Provide conditions for all visitors to perform frequent hand hygiene with alcohol-based gel or by thoroughly washing their hands with water and soap at healthcare facility entrances, waiting rooms, and patient check-ins.
  Screen all patients and companions entering the urodynamic unit for signs and symptoms of COVID-19 (i.e. fever, cough, myalgia, or fatigue (12)) as well as known exposures. Perform temperature measurement to check if patient or companions have fever (> 37.8 C). Reschedule the appointment if needed. Patients and companions must use mask during all the time. Spare masks must be available.
- During the exam.
  Standard precautions, including appropriate hand and respiratory hygiene, personal protective equipment (PPE) use, appropriate waste management, environmental cleaning, and patient-care equipment sterilization procedures should always be followed.
  Only the patient and essential staff should be present during urodynamic procedures. Companions should be avoided whenever possible. Two meters distance should be kept between the patient and staff, except for procedures such as catheterization and examination of the patient (7, 15).

### PPE for patients and companions

Patients must always wear a face mask. They should perform hand hygiene when entering and leaving the procedure room, by using alcohol hand-gel or thoroughly washing their hands for at least 20 seconds (7, 8). The same instructions apply to a companion, if absolutely needed during the test.

### PPE for the examiner and staff

The proper use of PPE is the most important method to avoid contamination of health care professionals. Single-use gloves, aprons and masks are recommended during urodynamic evaluation for all the staff. Because the test may involve body fluids, contact and coughs, eye protection with a face visor or protection glasses should also be used (7, 8, 13). Gloves, aprons and masks should be discarded after the exam. Face visors and protection glasses must be disinfected with 70% liquid alcohol (15).

### Stress incontinence tests

Maneuvers to increase abdominal pressure either to confirm signal quality or to provoke stress urinary leakage should be performed through straining (7). Since coughing results in airborne particles, it should be avoided whenever possible. Instead, Valsalva maneuvers should be used. If strictly necessary, cough must be directed away from the staff and with the protection of the elbow or a handheld tissue which must be discarded promptly. The mask must not be touched. Since urinary leakage must be detected by the examiner, female patients should be standing or squatting during stress maneuvers to allow leakage to be seen from distance. During video urodynamics this is not necessary since the fluoroscopic images may confirm urinary leakage.

## COMMENTS

Urodynamics is an essential diagnostic tool in the evaluation of lower urinary tract dysfunction. After a few months when these tests were virtually deleted from urological practice due to the COVID-19 pandemic, a slow return towards the reestablishment of urodynamic practice is on the way. This process needs adaptations and medical societies are urged to provide guidance. Our current recommendations on good urodynamic practices during the COVID-19 pandemic are mainly based on the risk stratification system proposed for surgical procedures (7, 11). In the last few months, expert opinion-based criteria have been validated by international medical societies, prioritizing high-risk patients for upper urinary tract deterioration but also considering factors such as current coronavirus spread rate, flexibilization of social distancing, availability of human resources and capacity of local health systems. SARS-COV-2 prevention and control recommendations regarding urodynamic practice are organized into two different levels, including pre-urodynamic and peri-urodynamic adaptations.

Pre-urodynamic recommendations start by prioritizing patients for examination based on the risk for upper urinary tract deterioration and whether performing the urodynamic test would alter the current treatment of the patient, especially if it may involve a surgical procedure.

Other important measures include reducing the number of daily performed exams to ensure physical distancing, ample use of phone contact to reduce the length of stay in the urodynamic unit and to screen for symptoms of infection (i.e., fever, cough, fatigue, myalgia) as well as known exposure to COVID-19 patients.

Peri-urodynamic measures comprise proper use of personal protective equipment for both patients and health-care professionals, maintaining ample physical distance and avoiding cough maneuvers during urodynamic tests.

It is important to emphasize that given the dynamic evolution of the pandemic as well as the regional differences in our country, the urodynamic practitioner should reconcile the current recommendations to the local situation.
